# Psychological Adjustment of Healthcare Workers in Italy during the COVID-19 Pandemic: Differences in Stress, Anxiety, Depression, Burnout, Secondary Trauma, and Compassion Satisfaction between Frontline and Non-Frontline Professionals

**DOI:** 10.3390/ijerph17228358

**Published:** 2020-11-12

**Authors:** Carmen Trumello, Sonia Monique Bramanti, Giulia Ballarotto, Carla Candelori, Luca Cerniglia, Silvia Cimino, Monia Crudele, Lucia Lombardi, Silvia Pignataro, Maria Luisa Viceconti, Alessandra Babore

**Affiliations:** 1 Department of Psychological, Health and Territorial Sciences, University “G. d’Annunzio” of Chieti, via dei Vestini, 66100 Chieti, Italy; c.trumello@unich.it (C.T.); sonia.monique92@gmail.com (S.M.B.); c.candelori@unich.it (C.C.); monia.crudele@studenti.unipd.it (M.C.); lucia.lombardi@unich.it (L.L.); silvia.pignataro@studenti.unich.it (S.P.); marialuisa.viceconti@studenti.unich.it (M.L.V.); 2 Dipartimento di Psicologia Dinamica e Clinica, “Sapienza” Università di Roma, 00185 Roma, Italy; giulia.ballarotto@uniroma1.it (G.B.); silvia.cimino@uniroma1.it (S.C.); 3 Facoltà di Psicologia, Università Telematica Internazionale Uninettuno di Roma, 00186 Roma, Italy; l.cerniglia@uninettunouniversity.net

**Keywords:** COVID-19, healthcare workers, anxiety, depression, stress, burnout, compassion satisfaction, secondary trauma, psychological support, pandemic

## Abstract

Emergency situations have been associated with negative psychological adjustment outcomes in healthcare professionals, although studies on the impact of the Coronavirus Disease 2019 (COVID-19) pandemic amongst Italian health workers are limited. The main aim of this study was to investigate the psychological adjustment of healthcare professionals during the peak of the COVID-19 pandemic, evaluating differences according to working or not with patients affected by COVID-19 and in areas with a more severe spread of this pandemic. Healthcare professionals’ attitudes toward psychological support were analyzed. The levels of anxiety, depression, psychological stress, and professional quality of life (compassion satisfaction, burnout, and compassion fatigue) and attitudes toward psychological support were measured among 627 Italian healthcare workers (mean age = 40.55 years; SD = 11.49; range: 27–72). Significantly higher levels of stress, burnout, secondary trauma, anxiety, and depression were observed among professionals working with COVID-19 patients. Higher levels of stress and burnout and lower levels of compassion satisfaction were detected in professionals working in areas with higher rates of contagion. No interaction effects were found between working (or not) with patients affected by COVID-19 and working (or not) in areas with a more severe diffusion of this pandemic. Finally, in the group of professionals who worked with COVID-19 patients, the percentage of professionals who thought to ask for psychological support was twice that of the group that did not work with COVID-19 patients. The overall findings indicate that the mental health of frontline healthcare workers requires further consideration and that targeted prevention and intervention programs are necessary.

## 1. Introduction

At the end of December 2019, the Severe Acute Respiratory Syndrome Coronavirus 2 (SARS-CoV-2), then renamed Coronavirus Disease 2019 (COVID-19) by the World Health Organization [1], was first identified in China. COVID-19 rapidly spread all over the world, and the WHO declared a pandemic state on 11 March 2020. At the time of the revision of this manuscript (October 2020), more than 40 million people have been infected [2].

In Italy, the first epidemic outbreak was confirmed in February 2020 [3]; since then, thousands of cases have occurred, and, during the data collection for the current research (mid-April), the most affected regions were Lombardy, Piedmont, Emilia Romagna, and Veneto, each one with more than 10,000 cases of infected individuals [2]. Facing this critical situation, the Italian government implemented measures to reduce the local transmission of the virus and promulgated a lockdown throughout the entire country. This emergency changed everyone’s lives and work habits, especially those of the healthcare workers, who play the most important role in public health [4]. They were directly involved in dealing with this critical condition, which exposed them to a major risk of becoming infected, major psychological pressure related to uncertainty about the duration of the crisis, a lack of proven therapies or a vaccine, and potential shortages of healthcare resources, including personal protective equipment [5,6]. In addition, healthcare workers have often chosen to live far from their families to protect them from the risk of contagion during this period [5].

Previous literature focused on the psychological risks of healthcare workers related to other epidemics, detecting high levels of anxiety, depression [7,8], stress, and burnout [8,9]. Symptoms such as anxiety and fear have immediately increased in the early stages of an epidemiological crisis but rapidly decreased in the later stages, whereas depression and post-traumatic stress symptoms have persisted over time [10]. Frontline healthcare workers involved in the diagnosis, treatment, and care of COVID-19 patients have reported more severe symptoms of anxiety, depression, and stress than those not on the front line [6,11].

The literature on emergency work has focused on the effects of working in critical situations, especially on burnout, as it poses a risk for healthcare workers [9,12]. As a concept, burnout refers to the emotional affect associated with feelings of frustration and powerlessness that develop in professionals’ negative attitudes at work [13,14]. Individuals who work in healthcare services have been identified as a high-risk group for burnout, and the workers who have symptoms of this syndrome are less satisfied with their work activities than others [15,16]. In a recent study in this population, more than 62% of workers showed a moderate degree of burnout [17].

With these theoretical premises in mind, we thought it important to measure the professional quality of life in the current emergency context. Figley [18,19] proposed two relevant aspects: compassion fatigue and compassion satisfaction. Compassion fatigue [12,18] is an outcome of prolonged and intense contact with patients and exposure to continuous stress; it may overlap with the concept of secondary trauma [9,20,21]. Figley [18] extended this concept to a combination of behavioral and emotional reactions following attempts to aid traumatized people. Compassion satisfaction represents the positive aspect of being a healthcare worker, related to an empathetic attitude and inclination to take care of suffering patients [9,22,23]. These factors play a fundamental role in professional quality of life [24,25,26]; stress and negative affects seem to be positively correlated with compassion fatigue, whereas positive affect and personal/social factors are positively correlated with compassion satisfaction [9]. According to Stamm [27], compassion fatigue incorporates two aspects: burnout (exhaustion, frustration, anger, and depression) and secondary trauma (negative feelings driven by fear and work-related trauma). In this paper, we refer to compassion fatigue considering its two aspects: burnout and secondary trauma.

To summarize, emergency situations, such as the current COVID-19 pandemic, have been associated with both psychological adjustment and professional quality of life outcomes.

### Study Aims and Hypotheses

According to these premises, we conducted a study in a sample of healthcare workers in Italy. To the best of our knowledge, this is among the first studies in Italy that have investigated the psychological impact of the pandemic on healthcare workers. Specifically, our main aim was to analyze the psychological adjustment of Italian healthcare professionals during the peak of the COVID-19 pandemic in terms of perceived stress, anxiety, depression, and professional quality of life (i.e., burnout, secondary trauma, and compassion satisfaction). More specifically, we examined the differences in these variables according to (1) working or not working with patients affected by COVID-19, and (2) working or not working in areas with a more severe diffusion of this pandemic. According to the Ministry of Health, Italian regions with more than 10,000 infected individuals at the peak of the first wave were defined as the most critical (i.e., Lombardy, Piedmont, Emilia Romagna, and Veneto). Following previous research amongst Chinese healthcare workers [6,11,28,29], we hypothesized that healthcare professionals working with patients affected by COVID-19 and in the most affected Italian regions would report higher levels of distress than the other categories (i.e., not working with COVID-19 patients or living in other regions). Previous literature on emergency situations merged these two conditions (working with affected patients and working in areas with a more severe diffusion of the pandemic) into a single group of frontline workers. We preferred to keep the two groups separate to better observe differences between them. Finally, we aimed to explore healthcare professionals’ attitudes toward psychological support by asking them whether they have considered asking for assistance, according to working or not with COVID-19 patients and in the most affected regions.

## 2. Materials and Methods

### 2.1. Participants

The participants comprised 627 healthcare workers, with a mean age of 40.55 years (SD = 11.49; range: 27–72). The descriptive statistics of the participants’ characteristics are presented in Table 1. The participants had been working as healthcare professionals for 13.76 years on average (SD = 11.39). The majority of the sample had not been infected by COVID-19 (93.9%); only a small percentage was infected, with (1.2%) or without (4.9%) symptoms. To verify differences in psychological adjustment between healthcare workers infected by COVID-19 and not infected, the Mann–Whitney U test was carried out. Differences in the distributions of the two groups were found relating to perceived stress (*p* < 0.05), anxiety (*p* < 0.01), and depression (*p* < 0.05), with infected healthcare workers showing worse psychological adjustment than their colleagues. As for marital status, most of them (75.4%) had a current relationship; the remainder (24.6%) were divorced, widowers, or single, and 50.1% had at least one child.

At the time of questionnaire administration, 48.8% were working with COVID-19 patients and 33.5% of the study participants were working in the most affected Italian regions (regions with more than 10,000 infected individuals as defined by the Italian Health Ministry).

### 2.2. Measures

#### 2.2.1. Sociodemographic Information

To gather sociodemographic information, an ad hoc questionnaire was prepared that consisted of questions regarding personal information (e.g., sex, age, nationality, and household income), work (e.g., years of working and the type of healthcare profession), and a question about the attitude toward psychological support (no, I do not think I need it; I thought about it but I do not think I will start it; I think I will but I have not started yet; I have already started sessions of psychological support).

#### 2.2.2. Hospital Anxiety and Depression Scale

The Hospital Anxiety and Depression Scale (HADS) [30,31] is a reliable self-report instrument that consists of two independent subscales: anxiety (HADS-A; sample items: “I get a sort of frightened feeling as if something awful is about to happen” and “Worrying thoughts go through my mind”) and depression (HADS-D; sample items: “I have lost interest in my appearance” and “I look forward with enjoyment to things”), each containing seven items. Each item is scored on a 4-point Likert scale, with a total score between 0 and 21 for either anxiety or depression. In previous studies, this instrument showed good reliability [32], such as in our study, where the Cronbach’s α was 0.893.

#### 2.2.3. Perceived Stress Scale

The short version of the Perceived Stress Scale (PSS-10) [33,34] was used to measure perceived stress. It was developed by Cohen et al. [33] to assess the degree to which situations in one’s life are evaluated as stressful and comprises 10 items: 6 negatively stated (sample item: “In the last month, how often have you been upset because of something that happened unexpectedly?”) and 4 positively stated (sample item: “In the last month, how often have you felt confident about your ability to handle your personal problems?”), rated on a 5-point Likert scale. The total scores range from 0 to 40, with higher scores showing higher levels of perceived stress. In this study, the Cronbach’s α was very good (α = 0.883), similar to that in previous studies that used this same tool [35,36].

#### 2.2.4. Professional Quality of Life Scale

The perceived quality in relation to work as a helper was assessed using the Professional Quality of Life Scale-V (ProQOL-V) [37], which comprises 30 items corresponding to three subscales: compassion satisfaction (10 items), burnout (10 items), and secondary traumatic stress (10 items). The compassion satisfaction items are characterized by the positive and altruistic aspects of the helping work (sample item: “I get satisfaction from being able to help people”), unlike burnout (sample item: “I feel trapped by my job as a helper”) and secondary traumatic stress (sample item: “I think that I might have been affected by the traumatic stress of those I help”). Each item explores how often, during the previous month, the subject has experienced a series of emotional states because of the helping work. The responses are rated on a 5-point Likert scale, with higher scores on each subscale indicating higher secondary trauma, compassion satisfaction, and burnout. In our study, the value of Cronbach’s α was satisfactory (α = 0.724), in line with recent research [24].

### 2.3. Data Analysis

All the statistical analyses were carried out through the Statistical Package for the Social Sciences (IBM Corp; Released 2010; IBM SPSS Statistics for Windows, Version 19.0.; Armonk, NY: IBM Corp.). The significance level was set to *p* < 0.05. First, the skewness and kurtosis of all the variables were tested to check their distributions. To verify the existence of differences between groups in the psychological adjustment of healthcare workers, a multivariate analysis of variance (MANOVA) was carried out. The between-subject factors were working (or not) with patients affected by COVID-19 and working (or not) in areas with a more severe diffusion of this pandemic; the dependent variables were perceived stress, professional quality of life, anxiety, and depression. Finally, a chi-square analysis for categorical variables was conducted to explore healthcare professionals’ attitudes toward the need for psychological support during this emergency period caused by the COVID-19 pandemic.

### 2.4. Procedure

This study was a cross-sectional survey conducted on the psychological status of Italian healthcare professionals during the peak of the COVID-19 pandemic. To involve subjects from all the Italian regions, a web-based survey was sent on the Internet to several groups of healthcare workers through mainstream social media (WhatsApp, Facebook, etc.). Participants were recruited via snowball sampling and were also asked to forward the survey to others. The inclusion criteria were working as a healthcare professional, working in Italy, and being over 18 years of age. Data were collected from 11 to 16 April 2020 (six weekdays), and all 20 Italian regions were represented.

The participants were informed about the aim and the procedure of the study. They were requested to provide their informed consent before completing the questionnaires anonymously. The participation in this study was voluntary and was not compensated. The procedure and all the questionnaires used in this survey were fully compliant with the indications of the Declaration of Helsinki and with the Ethics Code of the Italian Board of Psychology (the regulatory Authority providing the national guidelines for research and clinical practice). The study protocol was reviewed by the Institutional Review Board of Psychology (IRBP; Protocol Number: 20013) of the Department of Psychological, Health and Territorial Sciences.

## 3. Results

The distributions of all the variables may be considered as acceptably normal since their skewness and kurtosis were between −1 and +1 [38].

### 3.1. Assessment of Psychological Adjustment of Healthcare Workers

To verify the differences between groups in terms of the psychological adjustment of healthcare workers, a MANOVA was carried out. The independent variables were working (or not) with patients affected by COVID-19 (“COVID Patients”) and working (or not) in areas with a more severe diffusion of this pandemic (“Most Affected Regions”); the dependent variables were perceived stress, professional quality of life, anxiety, and depression.

The results showed a significant effect of the independent variables COVID Patients (λ = 0.95; F_(6618)_ = 5.37; *p* = 0.000) and Most Affected Regions (λ = 0.97; F_(6618)_ = 2.70; *p* = 0.014), but not for their interaction (λ = 0.99; F_(6618)_ = 0.41; *p* = 0.869). The results of the univariate effects are shown in Table 2 and Table 3 for the independent variables COVID Patients and Most Affected Regions, respectively.

With regard to COVID Patients, we observed statistically significantly higher levels of stress, burnout, secondary trauma, anxiety, and depression among professionals working with COVID-19 patients. No differences were detected in compassion satisfaction.

With regard to Most Affected Regions, our findings highlight that healthcare professionals working in the Italian regions most affected by the COVID-19 pandemic presented higher levels of perceived stress and burnout and lower levels of compassion satisfaction compared with healthcare professionals working in the other regions. No differences for other variables (anxiety, depression, and secondary trauma) were observed.

### 3.2. Healthcare Professionals’ Attitudes toward the Need for Psychological Support

Finally, we aimed to explore healthcare professionals’ attitudes toward the need for psychological support during this emergency period caused by the COVID-19 pandemic. Chi-square analyses were conducted. Although no significant effects were found when exploring psychological support needs according to working (or not) in the Italian regions most affected by this pandemic, significant effects were found for psychological support needs according to professionals working with COVID-19 patients and professionals not working with such patients. These results are summarized in Table 4.

The results show that most of the overall sample did not consider asking for psychological help, but this percentage significantly decreased in the group of professionals who worked with COVID-19 patients. In this latter group, the percentage of professionals who thought to ask for psychological support was double that of the group that did not work with COVID-19 patients.

## 4. Discussion

The literature suggests that medical staff treating patients with COVID-19 report high levels of anxiety and low self-efficacy levels [39,40], and other research on Wuhan healthcare workers reported great vulnerability to stress, anxiety, and depression, suggesting that frontline healthcare workers should be closely monitored as a high-risk group for maladjustment [29]. Excessive stress and/or anxiety in the clinical context may affect performance and can compromise patient outcomes [41].

In this study, we explored the psychological adjustment of Italian healthcare professionals during the peak of the COVID-19 pandemic in terms of perceived stress, anxiety, depression, and quality of professional life (including the three sub-variables of compassion satisfaction, secondary trauma, and burnout). In particular, our purpose was to verify whether working with patients affected by COVID-19 was associated with higher scores in the above study variables; we were also interested in verifying if these scores were affected by working or not in areas with higher reported cases and deaths. As some Chinese studies suggested, healthcare professionals working with COVID-19 patients were more at risk of higher levels of stress, anxiety, and depression [6,26,28]. In Italy, a study on the general population reported high stress levels during the COVID-19 pandemic [42], whereas studies specifically addressing healthcare workers’ adjustment are increasing but still few in number [24,35,43].

To address our first objective, we examined the existence of differences in psychological assessment results according to (1) working or not with patients affected by COVID-19, and (2) working or not in areas more severely affected by this pandemic in terms of case count. The results showed significant differences between both these groups, but not for their interaction. Specifically, the findings showed no difference in compassion satisfaction and a significant difference in the distribution of perceived stress, anxiety, depression, burnout, and secondary trauma levels between healthcare workers who worked or not with patients affected by the COVID-19 disease. In particular, consistent with our hypothesis, healthcare workers directly involved with COVID-19 patients reported higher levels in all these variables than their colleagues working with non-COVID-19 patients. This result is in line with two recent Chinese studies [6,28] that considered a sample of frontline care workers, reporting a high prevalence of mental health symptoms among these professionals. In this sense, our study supports previous research but adds further knowledge about other key variables such as burnout and secondary trauma that were not addressed in previous research on COVID-19. In the literature on other pandemics, burnout was posited to be associated with stress, anxiety, depression, and secondary trauma in frontline workers [14], and some authors found that it might contribute to the increased incidence of their stress and depression [44,45]. Zhang et al. [9] demonstrated that burnout levels in medical staff and other healthcare workers were typically higher than in other populations, and Bansal et al. [46] stated that clinicians were more likely to show burnout symptoms than the general workforce, which decreased performance and quality of life. In general, the rates of burnout among healthcare workers are posited to be high [47]. Although several authors have evaluated burnout in frontline workers, our study is, to the best of our knowledge, the first to address it in the context of the COVID-19 pandemic in Italy.

The concept of secondary trauma is strictly associated with burnout, as both form compassion fatigue; more specifically, it refers to secondary exposure to people who have experienced extremely or traumatically stressful events [27]. As expected, healthcare professionals working with COVID-19 patients showed higher levels of secondary trauma than their colleagues, but this result conflicts with the findings of Li et al. [48], who reported higher levels of this variable among non-frontline nurses than among frontline ones. Following the authors’ suggestion, their data could be explained by the possible higher psychological endurance of frontline nurses [48]. In this sense, frontline medical workers could be more accustomed to potentially distressing experiences than non-frontline workers, therefore showing a lower negative response to challenging situations. However, this was not the case in our frontline sample, which showed higher levels of secondary trauma compared with non-frontline colleagues.

With regards to the differences between healthcare workers that carry out their work in the regions most heavily affected by COVID-19 and those working in less impacted areas, our findings highlight that healthcare professionals working in the Italian regions most affected by the COVID-19 pandemic presented higher levels of perceived stress and burnout and lower levels of compassion satisfaction than the other group. This result is consistent with previous studies analyzing interregional differences. Lai et al. [6], for instance, found higher levels of distress in medical staff in the most affected Chinese region (Hubei) and, specifically, in Wuhan [6]. Our study is amongst the first to address this finding in Italy, which suffered high rates of contagion throughout the country. On one of the last days of our data collection (15 April 2020), Italy was the first country in the world in terms of the number of deaths (21,647 in total from the beginning of the COVID-19 pandemic) and the second country (Spain was the first) for the number of infections (165,155 total cases) [1]. However, the rates of contagion showed relevant differences according to different Italian geographical areas. On the last day of our data collection, the most affected Italian region was Lombardy, with more than 63,000 cases, while the least affected regions were Molise and Basilicata, with about 300 cases each (www.protezionecivile.gov.it).

If we observe the differences between a) working or not with COVID-19 patients and b) working or not in the Italian regions with the higher infection rates and number of deaths, we notice some interesting elements. Overall, the former condition was associated with a worse psychological adjustment, as it presented higher levels of all the study variables (namely, stress, burnout, secondary trauma, anxiety and depression), with the exception of compassion satisfaction. The latter condition (working in the Italian regions with the higher rates of infected individuals and deaths) was only associated with higher stress and burnout.

Another interesting result was that contrary to our expectations, we found no significant interactions between these two variables (working or not with COVID-19 patients and working or not in the Italian regions with the higher infection rates and number of deaths). We suppose that other variables (such as the relationships that develop within the medical staff or the perceived social support) should be considered to explain why healthcare professionals working in the most critical situations (i.e., with COVID-19 patients and in the most affected Italian regions) had no worse psychological adjustment than the other categories of colleagues. However, as we did not address these variables, no conclusions can be drawn in this direction. Other research is needed in order to further explore these issues with larger and numerically homogeneous groups. Overall, our findings underline that, differently from previous literature on emergency situations that merged these two conditions (working with affected patients and working in areas with a more severe diffusion of the pandemic) into a single group of frontline workers [11,28,29], future studies should separate those two groups to more clearly observe their differences.

A further result from our study concerns compassion satisfaction, which refers to an empathetic attitude and inclination to take care of suffering patients [9,22]. In some studies, it was associated with positive affect, empathy, and high levels of professional quality of life [9,22,49]. Compassion satisfaction occurs in the form of the helper’s altruistic behaviors that are aimed at alleviating the patients’ suffering [50]; healthcare workers experiencing high levels of compassion satisfaction were able to provide competent and compassionate care [50]. This construct was identified as a factor protective against burnout and compassion fatigue in previous studies [51]. In our results, no differences were detected between professionals working or not with COVID-19 patients, whereas it was significantly lower in professionals working in the Italian regions most affected by the current pandemic. We may explain this apparently contradictory result as a consequence of the particularly difficult conditions in which healthcare professionals worked in the most affected regions during the peak of pandemic: a fundamental basis of compassion satisfaction is constituted of an empathetic relationship with the patient’s family and support from colleagues [49]. All these aspects were likely compromised by working in the Italian regions most affected by the COVID-19 pandemic but not in the other regions when also working with COVID-19 patients. However, this result has to be considered cautiously; future research is needed to explore this finding.

Finally, we investigated healthcare professionals’ attitudes toward the need for psychological support during this emergency period. We observed that most of the total sample did not consider asking for psychological help, but this percentage was significantly lower in the group of professionals who worked with COVID-19 patients. This finding could be explained by the very difficult conditions in which they lived, from both personal and employment viewpoints. They had to deal with overwork, a high risk of infection, and, sometimes, inadequate protection from contamination, frustration, high-risk patients with negative feelings, discrimination, isolation, a lack of contact with their families, and exhaustion [11]. It was not surprising that they thought of asking for psychological help. However, caution is needed in drawing conclusions, as this question does not highlight if individuals currently receiving psychological support had actually started it before the COVID-19 pandemic, and if those who were considering support were doing so because of their work or because of other personal factors. Therefore, these issues should be analyzed in future studies.

The limitations of this study include the fact that the sample size was unbalanced for sex, with women representing the majority, although this distribution is representative of the sex differences among Italian healthcare workers [52]; the number of participants at the intersection of the two between-subject factors we considered (working or not with COVID-19 patients and working or not in the Italian regions with the higher infection rates and number of deaths) was not balanced; our measurements were exclusively based on self-report questionnaires; and the cross-sectional design did not allow drawing cause–effect relationships. All these aspects limit the generalizability of the current research to other professional groups and situations different from the current COVID-19 pandemic.

Notwithstanding these limitations, our study offers some suggestions about health professionals’ psychological adjustment during the climax of the COVID-19 pandemic. This category of workers has been working on the frontline during the outbreak, facing extreme conditions that have never occurred before, at least in Italy, with isolation from family and friends, social distancing, and shifts requiring intense work. No proven therapies or vaccines were available during the first wave of the pandemic. We hypothesize that these conditions found healthcare professionals unprepared to deal with them; despite this, all the healthcare workers faced this exceptional situation, which in some cases led them to become infected with COVID-19. In this group, we observed higher levels of stress, anxiety and depression when compared with their non-infected colleagues, but, as the percentage of infected workers in our sample was very low, further research is necessary to more deeply explore this issue.

As future pandemics may be more frequent than in the past [53], it is important to implement support programs specifically dedicated to medical staff. Protecting the mental health of healthcare workers is an important issue for public health and policymakers. If pandemics are disastrous events affecting the entire population, they especially impact healthcare systems (e.g., inadequate resources for medical responses, healthcare worker illness, fewer services for the population, etc.), having important social and economic repercussions [54]. In this difficult situation, policymakers could increase hiring in this job sector, encourage more flexible work shifts, and implement psycho-social support for this category of workers. Therefore, health authorities should not only improve working conditions but also plan specific intervention and support services to reduce the psychological burden on health workers. Among these specific interventions, psychological support should be delivered remotely (by the Internet or phone) and scheduled to adapt to the work shifts of the professionals.

## 5. Conclusions

In our study, to the best of our knowledge, we were amongst the first to examine the psychological adjustment of Italian healthcare professionals during the peak of the current COVID-19 pandemic, considering a number of variables referring to personal and professional adjustment. This category of professionals was the one most directly involved in managing the emergency caused by the very rapid spread of the SARS-CoV-2 pandemic in Italy, which produced a high number of deaths and individuals infected that were in serious medical conditions (on 15 April 2020 in Italy, there were 27,643 individuals hospitalized with symptoms and 3079 hospitalized in intensive care units [55]). Overall, our findings highlight that professionals working with COVID-19 patients are at higher risk of stress, burnout, secondary trauma, anxiety, and depression, and health professionals working in the most affected areas are at a major risk of stress, burnout, and low compassion satisfaction. We also found that in the group of professionals who worked with COVID-19 patients, the percentage of professionals who thought to ask for psychological support was double that of the group that did not work with such patients. This last topic has never been addressed before during the current COVID-19 pandemic. Taken together, these results indicate that the mental health of frontline health workers requires further consideration and that targeted prevention and intervention programs are necessary. If, as it seems, we will have to acclimate to living with the risk of pandemics in our future, it is certainly important to be prepared, especially by supporting the professionals who will work on the front line.

## Figures and Tables

**Table 1:** Demographic characteristics of the study sample (*N* = 627).

Sample Characteristics		*N* (%)
Sex	Male Female	125 (19.9) 502 (80.1)
Nationality	Italian Foreign	608 (97) 19 (3)
Household income (EUR/year)	0–15,000 15,001–28,000 28,001–55,000 55,001–75,000 >75,000	56 (8.9) 235 (37.5) 220 (35.1) 65 (10.4) 51 (8.1)
Working with COVID-19 patients *N* = 306 (48.8%)	Working in regions with higher rates of contagion and deaths *N* = 210 (33.5%)	131 (42.8)
	Working in other regions *N* = 417 (66.5%)	175 (57.2)
Working without COVID-19 patients *N* = 321 (51.2%)	Working in regions with higher rates of contagion and deaths *N* = 210 (33.5%)	79 (24.6)
	Working in other regions *N* = 417 (66.5%)	242 (75.4)

**Table 2:** Mean differences between healthcare professionals working or not working with COVID-19 patients.

	Not Working with COVID-19 Patients Mean (SD)	Working with COVID-19 Patients Mean (SD)	F_(1,623)_	Partial Eta Squared
Perceived Stress	17.82 (7.27)	19.78 (6.88)	8.47 **	0.013
Burnout	26.38 (6.76)	29.70 (7.35)	24.01 ***	0.037
Secondary Trauma	23.95 (7.23)	26.96 (8.58)	18.74 ***	0.029
Compassion Satisfaction	45.36 (8.79)	44.63 (8.15)	0.17	0.000
Anxiety	7.89 (4.28)	8.95 (4.14)	8.59 **	0.014
Depression	6.24 (4.02)	7.37 (4.06)	8.51 **	0.013

Note: ** *p* ≤ 0.01; *** *p* ≤ 0.001.

**Table 3:** Mean differences between healthcare professionals working or not working in the most affected Italian regions.

	Not Working in the Most Affected Regions Mean (SD)	Working in the Most Affected Regions Mean (SD)	F_(1623)_	Partial Eta Squared
Perceived Stress	18.07 (7.12)	20.18 (6.99)	7.93 **	0.013
Burnout	27.33 (7.05)	29.33 (7.46)	5.30 *	0.008
Secondary Trauma	25.13 (8.20)	25.99 (7.76)	0.12	0.000
Compassion Satisfaction	45.59 (8.28)	43.85 (8.79)	5.28 *	0.008
Anxiety	8.22 (4.21)	8.80 (4.29)	0.91	0.001
Depression	6.57 (3.97)	7.22 (4.25)	1.60	0.003

Note: * *p* ≤ 0.05; ** *p* ≤ 0.01.

**Table 4:** Distributions of the variables levels between healthcare professionals working or not working with COVID-19 patients.

Variables			Not Working with COVID-19 Patients (*N* = 321) *N* (%)	Working with COVID-19 Patients (*N* = 306) *N* (%)	Total Sample *N* (%)	Sig.
About the possibility of seeking psychological support	I do not need it ^†^	*N*	216 (67.3%)	135 (44.1%)	351 (56.0%)	χ^2^_(2 df)_ = 36.041 ***
		Expected values	179.7	171.3		
	I considered it but I do not think I will start ^†^	*N*	67 (20.9%)	95 (31.0%)	162 (25.8%)	
		Expected values	82.9	79.1		
	I think I will ask for it but I have not started yet ^†^	*N*	27 (8.4%)	57 (18.6%)	84 (13.4%)	
		Expected values	43	41		
	I have already started it	*N*	11 (3.4%)	19 (6.2%)	30 (4.8%)	
		Expected values	15.4	14.6		
		*N*	321	306	627	

Note: *** *p* ≤ 0.001. ^†^ adjusted standardized residual ≥±2.
